# Hepatitis A, B, and C in Brazilian Afro-Descendant Communities from Northeast Brazil: A Seroepidemiological Survey

**DOI:** 10.3390/v16111652

**Published:** 2024-10-23

**Authors:** Barbara V. Lago, Aline B. Cardoso, Giselle P. Nascimento, Edvan Pereira, Rony A. Oliveira, Mônica de Avelar Figueiredo Mafra Magalhães, Juliana C. Miguel, Filipe Anibal Carvalho-Costa, Jacenir Reis dos Santos-Malett, Jurema Corrêa Da Mota, Francisco Inácio Bastos, Livia Melo Villar

**Affiliations:** 1 Laboratory of Viral Hepatitis, Oswaldo Cruz Institute, Oswaldo Cruz Foundation, Rio de Janeiro 21040-360, RJ, Brazil; allinne1325@hotmail.com (A.B.C.); g.nprado@hotmail.com (G.P.N.); julicm@ioc.fiocruz.br (J.C.M.); 2 Federal Institute of Piauí, São João do Piauí 64760-000, PI, Brazil; bioedvanpereira@gmail.com (E.P.); ronyao60@gmail.com (R.A.O.); 3 Laboratory of Health Information, ICICT, Oswaldo Cruz Foundation, Rio de Janeiro 21040-360, RJ, Brazil; monica.magalhaes@fiocruz.br (M.d.A.F.M.M.); correamota@gmail.com (J.C.D.M.); francisco.inacio.bastos@hotmail.com (F.I.B.); 4 Laboratory of Epidemiology and Molecular Systematics, Oswaldo Cruz Institute, Oswaldo Cruz Foundation, Rio de Janeiro 21040-360, RJ, Brazil; carvalhocosta70@hotmail.com (F.A.C.-C.); jacemallet@gmail.com (J.R.d.S.-M.)

**Keywords:** hepatitis, seroprevalence, Afro-Brazilian, HAV, HBV, HCV

## Abstract

Background: Viral hepatitis is a disease that is more prevalent among individuals residing in remote regions and in contexts of social vulnerability. The objective of this study was to ascertain the seroprevalence of hepatitis A (HAV), B (HBV), and C (HCV) in vulnerable communities in the rural area of São João do Piauí (SJP), northern Brazil. Methods: Immunoenzymatic assays were employed to detect the presence of anti-HAV (total and IgM), HBsAg, anti-HBc, anti-HBs, and anti-HCV serological markers in serum samples. Samples that yielded positive results were subjected to further analysis using real-time polymerase chain reaction (PCR). Results: A total of 492 individuals, ranging in age from 3 to 101 years, were consecutively recruited from eight rural communities. The majority of individuals were female (51.2%), over 30 years of age (57.1%), self-identified as Black/Brown (92.2%), and resided in Afro-Brazilian communities, designated as “quilombos” (68.1%). The seroprevalence of anti-HAV was 69.5% (95% CI: 65.4–73.6%), while that of anti-HBc was 4.7% (95% CI: 2.8–6.6%), and that of anti-HBs was 35.2% (95% CI: 30.1–39.4%), and 0.2% (95% CI:0.0–0.6%) for anti-HCV. Conclusions: The seroprevalence rates observed were higher than the national average, and a significant proportion of individuals in the target age groups were susceptible to HBV, despite the availability of vaccination. These findings highlight potential shortcomings in the management of vaccine-preventable diseases, which could have implications for public health.

## 1. Introduction

Hepatitis is a liver disease that may have severe consequences, including the development of cirrhosis and cancer. Globally, the most prevalent causes of viral hepatitis are the hepatitis A (HAV), B (HBV), and C (HCV) viruses. Despite their shared capacity to infect the liver, these viruses exhibit significant divergence in terms of taxonomic classification, modes of transmission, prevention strategies, severity of liver disease, progression to chronicity, and geographical distribution [1].

According to the World Health Organization (WHO), the global prevalence of viral hepatitis is approximately 400 million people, accounting for an annual mortality rate of up to 1.4 million people [1,2]. It is estimated that 4.5 million deaths could be prevented by 2030 in low- and middle-income countries through the adoption of effective interventions such as high-coverage vaccination, health education, and broader access to diagnosis and treatment [1].

Although Brazilian population-based studies have pointed to a decrease in viral hepatitis prevalence in recent decades, these infections remain a major challenge in hard-to-reach populations [3,4,5]. Despite the availability of HAV immunization for infants under 5 years old and HBV vaccine to all individuals regardless of age or risk group, vaccine coverage is still a public health problem in remote places [6]. Likewise, access to diagnosis in these places is far from optimal. Many carriers are unaware of their serological status until they develop severe liver disease, leading to human and financial losses [7,8,9]. Furthermore, these undiagnosed carriers are important viral reservoirs, playing a role in the chain of transmission.

Brazil is home to the largest population of individuals with African ancestry outside the continent of Africa. The majority of these individuals are descendants of Africans who were forcibly migrated to Brazil through the transatlantic slave trade. “Quilombos” (resembling “maroon settlements” in other contexts, but without the pejorative connotations associated with buccaneers or criminals) were locations offering refuge to fugitive slaves situated in remote and inaccessible areas. Remnant quilombo communities have persisted over time and are currently composed of “quilombola” Afro-Brazilian individuals, who are the descendants of the former community members [10]. This population continues to experience ethnic–racial inequalities, including limited access to health systems and low educational attainment [11,12]. To better design interventions to meet the WHO target of eliminating viral hepatitis, it is crucial to investigate the prevalence and vulnerabilities associated with viral hepatitis in hard-to-reach groups such as quilombolas and rural populations. The objective of this study was to ascertain the prevalence of seropositivity and active hepatitis A, B, and C virus infections in vulnerable communities in the rural area of São João do Piauí, northeast Brazil.

## 2. Materials and Methods

### 2.1. Study Population

This cross-sectional serosurvey was conducted from June to October 2019 in eight communities belonging to the rural area of the São João do Piauí (SJP) city, located in the State of Piauí, northeast Brazil. SJP has approximately 21,400 inhabitants, spread over an area of 1527.497 km^2^ (IBGE, 2022). Individuals from rural communities, namely, Eugênio, Bom Sucesso, Grajau, and Curtume, and remnant “quilombo” communities, namely, Junco, Lagoa do Boqueirão, Riacho dos Negros, and Saco, were included. These locations were consecutively selected. Visits were made to all households to collect blood samples and for the application of the socioepidemiological questionnaire. Information on variables such as age, gender, and occupation, as well as variables usually associated (i.e., putative risk factors) with hepatitis A, B, and C infections were collected via a structure questionnaire that was used by the research team in a previous study [13].

#### 2.1.1. Ethical Aspects

The study was approved by the Research Ethics Committee of Fundação Oswaldo Cruz—Fiocruz/IOC, protocol code N°889.582. The participants were informed about the confidentiality of the information, the voluntary nature of their participation, and their right to withdraw from the study at any time. All individuals signed the informed consent form. For those under 18 years old, assent was obtained from parents or guardians.

#### 2.1.2. Serological Analyses

Serum samples were tested for hepatitis A (total anti-HAV—The Elecsys^®^ Anti-HAV II test, Roche Diagnostics, Germany and anti-HAV IgM—Elecsys Anti-HAV IgM), hepatitis B (anti-HBc—Murex anti-HBc total, Diasorin, Italy; anti-HBs—Anti-HBs SYM Solution, Symbiosys, Brazil and HBsAg—Monolisa™ HBsAg ULTRA), and hepatitis C (Murex anti-HCV, Diasorin, Italy) serological markers.

#### 2.1.3. Molecular Analyses

HBV-DNA was quantified using the Abbott Real Time HBV (Abbott Diagnostics, Des Moines, IA, USA) assay, following the manufacturer’s instructions. Anti-HCV-serum-reactive samples were submitted for Real-Time PCR Cobas Taqman HCV 2.0 (Roche Diagnostics, Pleasanton, CA, USA). Anti-HAV IgM-positive samples were submitted for qualitative PCR to detect HAV RNA as previously described [14,15].

#### 2.1.4. Data Analysis

The information collected from the questionnaire and the findings from the tests were keyed into an e-spreadsheet. The sociodemographic characteristics and vulnerabilities of the studied communities were described by proportion, and the prevalence of HBV and HAV serological markers was calculated by community with their respective 95% confidence intervals. The analyses were performed using the Statistical Package for the Social Sciences (SPSS for Windows, version 21.0).

#### 2.1.5. Georeferencing Analysis

Using a Geographic Information System environment, the areas of the communities were delimited using satellite images available in the ArcGIS 10.4 Basemap library. Information on the number of inhabitants and serum samples were integrated into these images. Based on the value of the general prevalence of the different antibodies in the communities, heat maps were created using the Kernel Estimator to show the areas with the highest or lowest prevalence of each serological marker. Information on whether or not the community was “quilombola” was included in the analysis.

## 3. Results

A total of 492 individuals aged 3 to 101 years from eight rural communities were included in this study. Most individuals were female (51.2%), were over 30 yearsold (57.1%), with an education level up to elementary school (81.7%), with an income in Brazilian Reais equivalent to less than USD200.00 per month (51.1%). The vast majority of interviewees self-declared to be Black/Brown (92.2%) and to live in “quilombola” communities (68.1%) (Table 1).

A significant portion of the individuals, 47.9%, reported alcohol consumption, while 43.9% reported having undergone vaccination against hepatitis B virus (HBV). Other potential risk factors, including tattooing, piercing, blood transfusion, hemodialysis, illicit drug use, and the sharing of sharp objects, were reported by a smaller proportion of individuals (Table 1).

**Figure 1:**
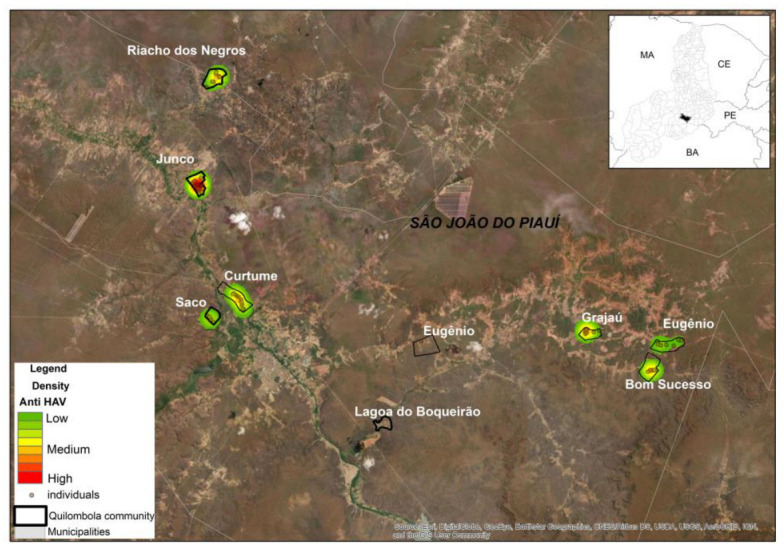
Kernel Estimator map of the total HAV antibodies.

The overall prevalence of HAV antibodies (total anti-HAV) was 69.5% (95% CI: 65.4–73.6%), indicating previous infection or vaccine-induced immunity. One individual tested positive for HAV IgM, with undetected HAV RNA. The communities with the highest and lowest prevalence rates were Lagoa do Boqueirão (93.7%; 95% CI: 80.4–100.0%) and Junco (53.2%; 95% CI: 44.3–62.3%), respectively (Table 2, Figure 1). All residents of the Lagoa do Boqueirão community participated in the study, and with the exception of one individual, all tested negative for anti-HAV.

**Figure 2:**
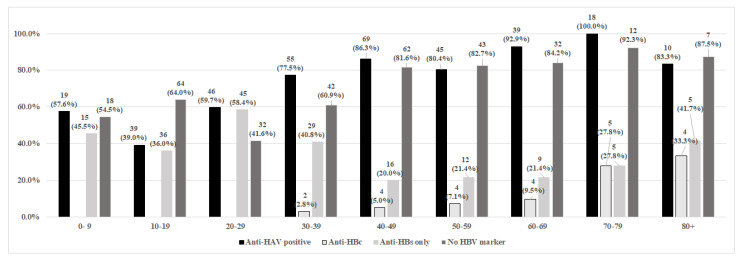
Data on current infections, past infections, susceptibility, and immunity consideringage of 492 participants, from the State Piauí, São João do Piauí, 2019–2020.

Figure 2 depicts the serological profile of the study participants stratified by age. Anti-HAV positivity was virtually 100% among individuals in the 70–79 age groups. The age group with the lowest anti-HAV positivity was composed of children and adolescents aged 10–19 (39.0%), followed by children aged 0–9 years old (57.6%).

With regard to hepatitis B, 4.7% (95% CI: 2.8–6.6) of individuals exhibited evidence of a past infection, as indicated by the presence of total anti-HBc (Table 2). Figure 2 shows that all individuals exposed to the hepatitis B virus (anti-HBc positive) were over 30 years old. The prevalence of exposure to HBV increased with age, with prevalences higher than 27% in participants aged 70 or above.

Four clusters of anti-HBc positivity, involving 12 out of 23 (52.2%) anti-HBc-positive individuals, were observed, which demonstrates the circulation of HBV among members of the same family in the past. One individual exhibited reactive serology for HBsAg (0.2%), accompanied by the detection of HBV DNA (viral load: 2.291 IU/mL). However, none of his family members tested positive for HBsAg or anti-HBc. All subjects reported having received the HBV vaccination and tested positive for anti-HBs.

The highest proportion of vaccine-induced immunity against HBV was observed in the age group of 20 to 29 years (58.6%). The 0–9 and 10–19 age groups exhibited anti-HB positivity rates of 45.5% and 36%, respectively (Figure 2). In total, 65% of individuals remain susceptible to HBV infection, including 64% of children and adolescents aged 10–19.

**Figure 3:**
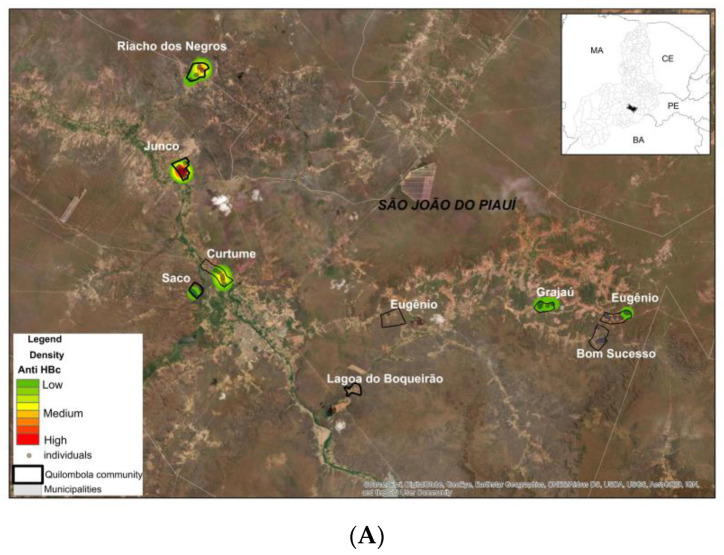

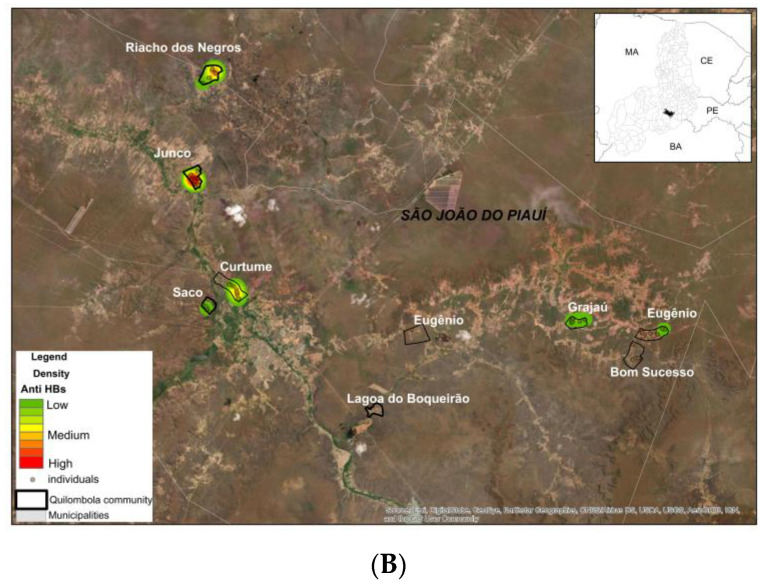
Kernel Estimator map of the Anti-HBc (**A**) and Anti-HBs (**B**).

A total of 173 individuals (35.2%) exhibited anti-HBs positivity, including 18 individuals who were also anti-HBc-reactive (18/173; 10.4%), indicating a history of infection with natural immunity. A serological profile compatible with vaccination was observed in only 155 of the 492 individuals (31.5%). It is noteworthy that the Bom Sucesso, Grajaú, Eugênio, and Saco communities exhibited a prevalence of susceptible individuals exceeding 70% (Table 2, Figure 3).

Anti-HCV was identified in a single participant (1/492; 0.2%), in conjunction with an undetected HCV RNA.

## 4. Discussion

Brazil is a country of continental dimensions, marked by pronounced geographic, economic, social, and ethnic inequalities. Consequently, certain diseases are more prevalent among individuals residing in remote regions and areas experiencing social vulnerability. Viral hepatitis is a socially determined disease, with a complex relationship to poverty, inadequate health information, and historical imbalances. The success of eradicating these diseases hinges on the implementation of structural public policies and comprehensive actions that are not only focused on early diagnosis and treatment, but also on improving social determinants such as sanitation, housing, and education [16,17].

Notwithstanding the endeavors of the Brazilian Ministry of Health to fulfill the WHO’s 2030 agenda, rural and remote communities continue to encounter impediments to access to diagnosis and vaccination, which are pivotal for attaining viral hepatitis elimination [16,17]. Effective micro-elimination actions are based on interventions at both ends of the transmission chain. On the one hand, this entails the accurate identification of carriers as viral reservoirs. On the other, it involves the immunization of susceptible individuals through free and accessible vaccination.

Hepatitis A can be transmitted through the ingestion of contaminated water and food. It is closely related to poor hygiene and deficiencies in water supply, waste collection, and sanitation [18]. A study conducted with the Brazilian urban population revealed that the prevalence of anti-HAV antibodies is heterogeneous across the country’s macro-regions. The least developed regions, namely, the north, northeast, and Midwest, exhibited the highest anti-HAV prevalences [19,20].

This study observed a global anti-HAV prevalence of 69.5%, which is similar to that reported in previous studies conducted in the same macro-region [19]. However, significant discrepancies were observed between the study communities. It is noteworthy that the communities of L. do Boqueirão, Bonsucesso, and Curtume exhibited anti-HAV prevalence rates exceeding 85%, a figure that surpasses the reported rates for this macro-region. In contrast, communities such as Junco and Riacho dos Negros exhibited prevalence rates of 53.2% and 55.9%, respectively, which are below the national average.

It is important to note, however, that serological tests are unable to distinguish vaccine antibodies from those resulting from past HAV infection. In Brazil, the hepatitis A vaccine has been incorporated into the National Immunization Program (PNI) for children aged 12–23 months since 2014, with an expansion to include children up to 5 years of age in 2017. In the community of Curtume, which is the closest to the urban area (4 km), all children born after 2014 tested positive for anti-HAV. Therefore, despite the high prevalence of HAV infection in this region, the elevated positivity for anti-HAV observed exclusively in this community may be partially attributed to the introduction of vaccination.

From a global and local perspective, the health status of a community is directly influenced by a multitude of factors, including environmental, social, and economic factors. As indicated by the United Nations Development Programme (UNDP), the city of São João do Piauí exhibits a Human Development Index (HDI) value of 0.645, which is classified as relatively low. However, communities that are difficult to access are even less developed, as evidenced by the limited access to treated water and sewage systems in the communities that are situated the greatest distance from the urban area. Furthermore, additional factors that may elucidate the discrepancies in anti-HAV prevalence between communities include the level of education attained by individuals, their personal hygiene practices, the number of residents per household, and the proximity to water sources such as rivers and lakes.

In this study, all individuals exposed to the hepatitis B virus (HBV) were over 30 years of age, with 74% of them being over 50 years old. As has been previously established, the probability of exposure to the hepatitis B virus (HBV) tends to increase with age. Older age groups have been shown to have a higher prevalence of individuals with serological evidence of past HBV infection [16,21]. This elevated prevalence may be attributed to a multitude of cumulative exposures, including the sharing of individual sharp objects, unprotected sexual practices, vertical and intra-family transmission during a period when vaccination was not universally accessible to all age groups, and other factors. It is noteworthy that intrafamilial transmission may have played a role in the past in the spread of hepatitis B virus (HBV) infection, as four familial foci of anti-HBc positivity were observed.

The positivity rate for isolated anti-HBs, a marker of anti-HBV vaccination, was 31.5%. The age group with the highest prevalence of individuals with a vaccination profile was those aged 20–29 years. Given that the hepatitis B vaccine is administered at birth and has been freely available since 1992, it was anticipated that the positivity rate would exceed 80% for individuals under 30 years of age. This is consistent with the 84% coverage observed in the state capital for children born in 2005 and the 86% national coverage reported by 2018 [21]. However, the findings of our study indicate that only 45.5% of individuals aged below 30 years old exhibited anti-HBs positivity. It is alarming that over 60% of children and adolescents aged 0–19 years old were susceptible to HBV, indicating significant deficiencies in immunization coverage. These gaps may be attributed to structural issues such as inadequate vaccine supply, low adherence to the three-dose vaccination schedule, and challenges in accessing health centers (which are predominantly located in urban areas). However, the possibility of an absence of an immune response in vaccinated individuals cannot be discounted.

However, the finding that no individual under the age of 30 was exposed to HBV may indicate that, despite vaccination coverage being much lower than expected, the HBV circulation in young adults has been reduced, potentially as a result of an indirect and beneficial effect of vaccination (i.e., the formation of clusters of community/herd immunity).

It should be noted that the present study was not without limitations. Despite our efforts to actively seek volunteers, the proportion of participants varied across communities, with coverage ranging from 50% to 100% of residents. While trained professionals visited homes to encourage participation, the number of volunteers in each community was not equivalent. Furthermore, some family members declined to participate in the study due to their absence from home during the data collection period, either due to work or educational commitments, or because of their refusal to provide a blood sample. The use of less invasive methods, such as dried blood collected by finger-prick or saliva collection, could be evaluated as a potential tool to overcome this limitation. As a result of these limitations, it was not feasible to obtain a comprehensive understanding of vaccination coverage across all communities. Furthermore, the study design does not allow for the determination of definitive cause-and-effect relationships.

It is acknowledged that suboptimal sampling and operational challenges may compromise statistical inferences. However, given the absence of a comprehensive mapping of all vulnerable communities and individuals, probability sampling is not a viable option [22].

The implementation of studies in impoverished contexts introduces additional complexities. It is acknowledged that the research was conducted in circumstances that may have an impact on the reliability of the statistical analysis. However, every effort was made to engage with an impoverished, underserved, and stigmatized community (in the case of the quilombolas, such conditions have been in place for over four centuries). Since the inception of the Atlantic slave trade in Brazil and the protracted and ineffective integration of former slaves into the broader community following the abolition of slavery in 1888.

It would be unwise to present our findings in an overly optimistic or naïve manner. The data revealed gaps in the immunization of children against hepatitis A and B viruses (HAV and HBV) in age groups that are virtually covered by the national immunization program (PNI) for childhood vaccination. This suggests that the vaccination coverage for other childhood diseases with a significant impact, such as poliomyelitis, measles, and meningitis, may also be below the levels recommended by the PNI.

The principal conclusion of our study is to underscore the imperative of facilitating comprehensive access to vaccination for rural and underserved populations, both from a public health standpoint and as a fundamental strategy for achieving the WHO’s 2030 objective of eliminating viral hepatitis and other infectious diseases.

Brazil should improve vaccination rates in remote areas, which requires a multifaceted approach that addresses logistical, social, and informational challenges. First, government should invest in mobile clinics equipped with medical professionals and necessary supplies to reach isolated communities. Healthcare vans, boats, or drones can be used, especially in the northeast Brazil and other areas with difficult terrain, to reach people living in geographically challenging areas. Another approach is the development of mobile apps or SMS-based systems that can help rural populations schedule vaccinations and receive reminders. This could ensure timely follow-ups for booster doses. In addition, digital health records could be implemented to track vaccinations in real time, ensuring accurate records and minimizing the chance of missed doses. The government should increase investment in local healthcare infrastructure to provide consistent vaccination availability. This could include training more local healthcare workers to administer vaccines and to strengthen cold chain systems (refrigeration for vaccines) to ensure that vaccines remain effective when transported to and stored in remote areas. Other strategies could include community engagement programs to collaborate with local leaders or quilombola communities to promote vaccine awareness and trust. Local leaders often have a strong influence and can help combat vaccine hesitancy. It is important to ensure that educational campaigns about vaccination are sensitive to cultural beliefs and communicated in local languages. Health education programs that specifically target vaccine misinformation and promote the benefits of immunization, tailored to rural and remote populations, are also important. Since internet access may be limited in remote areas, radio stations or village loudspeakers can be used to broadcast public service announcements. Other measures could include public–private partnerships working together with non-governmental organizations and private companies that have local experience in delivering health services to hard-to-reach areas; allow for flexible or weekend vaccination schedules to accommodate those who travel long distances or have inconsistent access to transportation; and partnerships with local transport services, such as buses or boats, to distribute vaccines or transport people from remote areas to centralized vaccination points.

These measures, when implemented together, could significantly improve vaccination rates in Brazil’s remote areas, leading to better public health outcomes.

## Figures and Tables

**Table 1:** Sociodemographic characteristics and vulnerabilities of the communities studied in São João do Piauí, June to October 2019.

Variables	N	%
Communities		
Rural	157	31.9
Rural “quilombola”	335	68.1
Gender		
Female	252	51.2
Male	240	48.8
Age		
Upto 30 yearsold	210	42.9
Over 30 yearsold	279	57.1
Education		
Up to elementary school	394	81.7
High school ormore	88	18.3
Ethnic group		
Black/Brown	404	92.2
White/other	34	7.8
Monthy income		
<USD 200	205	51.1
>USD 200	196	48.9
Potential risk factors		
Blood transfusion	22	4.4
Hemodialysis	7	1.4
Sharing sharp objects	72	14.6
Piercings	61	12.3
Tattoos	44	8.9
History of alcohol misuse	200	40.6
No treated water	52	10.5
No sewage	293	59.5
Use of illicit drugs	20	4.0
Vaccination against hepatitis B	216	43.9

**Table 2:** Prevalence of HBV and HAV serological markers in rural communities studied in São João do Piauí, June to October 2019.

Serological Profile		Communities								Total
		Bom Sucesso	Grajau	Eugênio	Curtume	Saco	R. dos Negros	Junco	L.	
									Boqueirão	
		*n* (%, 95% CI)	*n* (%, 95% CI)	*n* (%, 95% CI)	*n* (%, 95% CI)	*n* (%, 95% CI)	*n* (%, 95% CI)	*n* (%, 95% CI)	*n* (%, 95% CI)	*n* (%, 95% CI)
Participants/		29/51	67/151	61/156	64/205	65/155	68/74	122/247	16/16	492
total community (%)		(56.9)	(44.4)	(39.1)	(31.2)	(41.9)	(91.9)	(49.4)	(100.0)	100.0
HBV infection (HBsAg)	Positive	-	-	-	1 (1.6; 0.0–4.7)	-	-	-	-	1 (0.2; 0.0–0.6)
	Negative	29(100.0)	67 (100.0)	61 (100.0)	63 (98.4; 95.3–100.0)	65 (100.0)	68 (100.0)	122 (100.0)	16 (100.0)	491 (99.8, 99.4–100.0)
HBV exposure (anti-HBc)	Positive	-	2 (2.9; 0.0–7.2)	1 (1.6; 0.0–4.90)	4 (6.3; 0.15–12.3)	3 (4.6; 0.0–9.8)	5 (7.3; 0.9–13.7)	6 (4.9; 1.0–88.1)	2 (12.5; 0.0–30.7)	23 (4.7; 2.8–6.5)
	Negative	29 (100.0)	65 (97.1; 92.8–100.0)	60 (98.4; 95.0–100.0)	60 (93.8; 87.6–99.8)	62 (95.3; 90.1–100.0)	63 (92.7; 86.2–99.0)	116 (95.1; 91.1–99.0)	14 (87.569.3–100.0)	469 (95.3; 93.4–97.2)
HBV-susceptible (no marker)	Positive	23 (79.3; 63.6–95.0)	49 (75.4; 64.2–86.1)	48 (80.0; 69.6–90.4)	23 (38.3; 25.7–51.0)	45 (72.6; 61.2–84.0)	37 (58.7; 46.2–71.2)	79 (68.1; 59.5–76.7)	10 (71.4; 44.4–98.5)	314 (66.9; 62.7–71.2)
	Negative	6 (20.7; 5.00–36.4)	16 (24.6; 13.9–35.4)	12 (20.0; 9.6–30.4)	37 (61.7; 49.0–74.3)	17 (27.4; 16.0–38.8)	26 (41.3; 28.8–53.8)	37 (31.9; 32.3–40.5)	4 (28.6; 1.5–55.7)	155 (33.3; 28.8–37.3)
Vaccine-like profile (anti-HBs only)	Positive	6 (20.7;5.00–36.4)	18 (26.0; 16.0–37.78)	13 (21.3; 10.7–31.9)	40 (62.5; 50.3–75.0)	19 (29.2; 17.9–40.6)	30 (44.1; 32.0–56.2)	41 (33.6; 25.1–42.1)	6 (37.5; 10.8–64.1)	173 (35.2; 30.1–39.4)
	Negative	23 (79.3; 63.6–95.0)	49 (73.2; 62.2–84.0)	48 (78.7; 68.1–89.2)	24 (37.5; 25.3–49.7)	46 (70.8; 59.4–82.1)	38 (55.9; 43.7–68.0)	81 (66.4; 57.9–74.9)	10 (62.5; 35.9–89.1)	319 (64.8; 60.6–69.1)
Anti-HAV	Positive	25 (86.2; 72.8–99.5)	42 (62.6; 50.8–74.6)	43 (70.4; 58.7–82.3)	55 (85.9; 77.2–94.7)	49 (75.3; 64.6–86.1)	48 (70.5; 59.5–81.7)	65 (53.2; 44.3–62.3)	15 (93.7; 80.4–100.0)	342 (69.5; 65.4–73.5)
	Negative	4 (13.8; 4.44–27.1)	25 (37.4; 25.4–49.2)	18 (29.6; 17.7–41.3)	9 (14.1; 5.3–22.8)	16 (24.7; 13.9–35.4)	20 (29.5; 18.3–40.5)	57 (46.8; 37.7–55.7)	1 (2.7; 0.0–19.6)	150 (30.5; 26.4–34.6)

## Data Availability

All relevant data are presented in the manuscript.
